# Dense Cranial Electroacupuncture Stimulation for Major Depressive Disorder—A Single-Blind, Randomized, Controlled Study

**DOI:** 10.1371/journal.pone.0029651

**Published:** 2012-01-06

**Authors:** Zhang-Jin Zhang, Roger Ng, Sui Cheung Man, Tsui Yin Jade Li, Wendy Wong, Qing-Rong Tan, Hei Kiu Wong, Ka-Fai Chung, Man-Tak Wong, Wai-Kiu Alfert Tsang, Ka-chee Yip, Eric Ziea, Vivian Taam Wong

**Affiliations:** 1 School of Chinese Medicine, LKS Faculty of Medicine, The University of Hong Kong, Hong Kong, China; 2 Department of Psychiatry, Kowloon Hospital, Hong Kong, China; 3 Department of Psychiatry, Fourth Military Medical University, Xi'an, Shaanxi, China; 4 Department of Psychiatry, LKS Faculty of Medicine, The University of Hong Kong, Hong Kong, China; 5 Chinese Medicine Section, Hospital Authority, Hong Kong, China; Chiba University Center for Forensic Mental Health, Japan

## Abstract

**Background:**

Previous studies suggest that electroacupuncture possesses therapeutic benefits for depressive disorders. The purpose of this study was to determine whether dense cranial electroacupuncture stimulation (DCEAS) could enhance the antidepressant efficacy in the early phase of selective serotonin reuptake inhibitor (SSRI) treatment of major depressive disorder (MDD).

**Methods:**

In this single-blind, randomized, controlled study, patients with MDD were randomly assigned to 9-session DCEAS or noninvasive electroacupuncture (n-EA) control procedure in combination with fluoxetine (FLX) for 3 weeks. Clinical outcomes were measured using the 17-item Hamilton Depression Rating Scale (HAMD-17), Clinical Global Impression-severity (CGI-S), and Self-rating Depression Scale (SDS) as well as the response and remission rates.

**Results:**

Seventy-three patients were randomly assigned to n-EA (n = 35) and DCEAS (n = 38), of whom 34 in n-EA and 36 in DCEAS group were analyzed. DCEAS-treated patients displayed a significantly greater reduction from baseline in HAMD-17 scores at Day 3 through Day 21 and in SDS scores at Day 3 and Day 21 compared to patients receiving n-EA. DCEAS intervention also produced a higher rate of clinically significant response compared to n-EA procedure (19.4% (7/36) vs. 8.8% (3/34)). The incidence of adverse events was similar in the two groups.

**Conclusions:**

DCEAS is a safe and effective intervention that augments the antidepressant efficacy. It can be considered as an additional therapy in the early phase of SSRI treatment of depressed patients.

**Trial Registration:**

Controlled-Trials.com ISRCTN88008690

## Introduction

Although selective serotonin reuptake inhibitors (SSRIs) are the mainstay in the treatment of depressive disorders, the treatment outcomes are unsatisfactory [1]. There remains a large portion of depressed patients who cannot obtain a full remission and experience relapse and functional impairment [1], [2]. Moreover, the delay in the onset of the action of SSRIs prolongs patients' suffering and exposes them to great risk of suicide [3]. These shortcomings have led to a high demand for seeking alternative strategies that can enhance the antidepressant efficacy of SSRIs particularly in the early phase of the treatment [4].

Numerous studies and recent meta-analyses have shown that acupuncture is efficacious for various types of depressive disorders [5]–[7]. Although most acupuncture protocols used were developed from the doctrine of traditional Chinese medicine and empiricism rather than modern scientific rationale, experimental and clinical observations have found that electroacupuncture has robust immediate and short-term effects in alleviating pain, autonomic dysfunction, sleep, and mood symptoms [8]–[10]. This rapid effect is thought to be associated with the fast and direct modulation of multiple central neurochemical systems, especially the brainstem adrenalinergic (NA), serotonergic (5-HT) neuronal and hypothalamic neuroendocrine systems [10], which play the principal role in the pathophysiology of major depression [11]. These are the reasons to hypothesize that electroacupuncture can serve to enhance the antidepressant action in the early phase of SSRI treatment.

Dense cranial electroacupuncture stimulation (DCEAS) is a novel stimulation mode in which electrical stimulation is delivered on dense acupoints located on the forehead mainly innervated by the trigeminal nerve, efficiently modulating multiple central transmitter systems via the trigeminal sensory-brainstem NA and 5-HT neuronal pathways [12]. Several pilot studies have shown that DCEAS and similar approaches are effective in improving refractory obsessive-compulsive disorder (OCD) [12], major depressive disorder (MDD) [13], post-stroke depression [14], and MDD-associated residual insomnia [15].

Fluoxetine (FLX) is one of the most prescribed SSRIs for major depression worldwide [16]. This single-blind, randomized, controlled trial was designed to determine whether DCEAS intervention could produce greater clinical improvement compared to noninvasive electroacupuncture (n-EA) control procedure in the early phase of FLX treatment of patients with MDD.

## Methods

### Subjects

This single-blind, randomized, sham-acupuncture controlled trial was conducted in Department of Psychiatry at Kowloon Hospital of Hong Kong between August 2009 and March 2011. The study protocol was approved by Institutional Review Board (IRB) of the University of Hong Kong/Hospital Authority Hong Kong West Cluster and registered in www.controlled-trials.com (ISRCTN88008690). The protocol for this trial and supporting CONSORT checklist are available as supporting information (see Checklist S1 and Protocol S1).

Psychiatrists referred outpatients to the study. The inclusion criteria were: (1) age 25–65 years; (2) DSM-IV diagnosis of MDD [17]; (3) 17-item Hamilton Rating Scale for Depression (HAMD-17) score ≥18 [18]; and (4) Clinical Global Impression-Severity (CGI-S) score ≥4 [19]. Subjects were excluded if they had: (1) unstable medical conditions; (2) suicidal attempts or aggressive behavior; (3) a history of manic, hypomanic, or mixed episode; (4) a family history of bipolar or psychotic disorders; (5) a history of substance abuse within the previous 12 months; (6) investigational drug treatment in the previous 6 months; (7) current psychotropic treatment exceed one week; or (8) needle phobia. All participants gave voluntary, written and informed consent before entering the trial.

### Randomization and blinding

Patients were randomly assigned to either n-EA or DCEAS treatment at a ratio of 1∶1, using a random block scheme from an automatic computer program (SPSS version II). The assignment was done in a single-blind manner, in which the random codes were only known by the acupuncturists (W.W. and M. S.C.). The validity of the subject-blind design was ensured by sham acupuncture procedure performed on the forehead acupoints, which were outside the visual field of the subjects (see below). In order to minimize the expected effects, patients were not told about the potential response of control and DCEAS procedure during random assignment.

### Fluoxetine treatment

Unmedicated patients in both groups received orally administered FLX for 3 weeks in an open manner. FLX dose was initiated at 10 mg/day and escalated to an optimal dose within one week based on individual patients' response, with a maximum dose of 40 mg/day. Attaining a balance of efficacy and side effects, this FLX dosing regimen has been widely used in previous studies of major depression in the Chinese population [20], [21]. Those who were currently treated with FLX for no more than one week continued their FLX treatment with the same dose. Those who were currently treated with other psychotropic medications for no more than one week were required to be switched to the FLX regimen by gradually withdrawing the drugs within one week in order to wash out potential “carryover” effects. The information about the equivalent efficacy of FLX was offered to the patients. Concomitant use of other psychotropic drugs was not allowed. Medication compliance was determined by pill count at each study visit. Patients who required concomitant medications and those having less than 80% FLX compliance were advised to withdraw from the study.

### DCEAS and n-EA procedure

**Figure 1:**
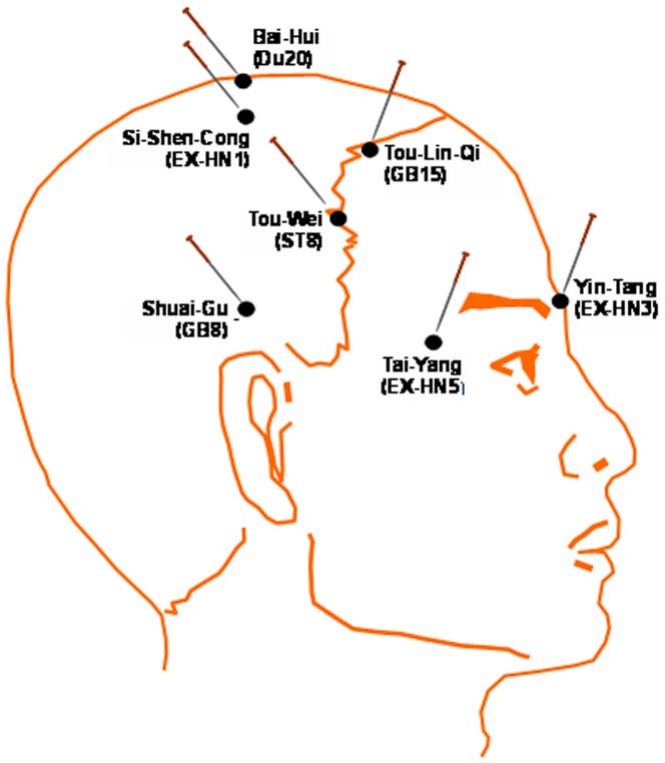
Acupoints used in dense cranial electroacupuncture stimulation (DCEAS).

The patients received 9 sessions of n-EA or DCEAS intervention (3 sessions per week) during FLX treatment. Electrical stimulation was delivered on the following 6 matches of forehead acupoints that are innervated by the trigeminal nerve via inserted or non-inserted needles (Fig. 1): Baihui (Du-20) and Yintang (EX-HN3), left Sishencong (EX-HN1) and Toulinqi (GB15), right Sishencong (EX-HN1) and Toulinqi (GB15), bilateral Shuaigu (GB8), bilateral Taiyang (EX-HN5), and bilateral Touwei (ST8). For DCEAS, disposable acupuncture needles (0.30 mm in diameter and 25–40 mm in length) were inserted into acupoints for a depth of 10–30 mm in a direction oblique or parallel to the surface. To ensure allocation concealment, the inserted needles were affixed with adhesive tapes so that DCEAS procedure was identical to control acupuncture procedure. Electrical stimulation with continuous waves at 2 Hz and constant current and voltage (9 V) was delivered via an acupuncture stimulation instrument (Hwarto, SDZ-II) for 30 min (the pulse width could not be determined in this model instrument). The choice of this stimulation mode was based on the fact that low frequency could exert broader effects on central neurochemical systems compared to high frequency and has been widely introduced into the treatment of neuropsychiatric disorders [10], [22]. The intensity of stimulation was adjusted to a level at which the patients felt most comfortable. For n-EA procedure, Streitberger's noninvasive acupuncture needles were used [23], [24]. Its validity and credibility have been well demonstrated [23], [24]. The needles with blunt tips were quickly put onto the same acupoints used in DCEAS without inserting into the skin. The needles were then affixed with plastic O-rings and adhesive tapes. Electrical stimulation was delivered with the same parameters as DCEAS. Patients felt the stimulation via blunt tips touched on the skin.

To ensure consistency in acupuncture procedure, the principal investigator (Z.J.Z.) provided a training workshop of acupuncture protocol. Acupuncture intervention was performed by registered acupuncturists (W.W. and S.C.M.) who had received 5-year undergraduate training in Chinese medicine and had practiced Chinese medicine over three years.

### Assessment

Treatment outcomes were changes from baseline in the total score on HAMD-17 [18], CGI-S [19], and the Chinese-version Self-rating Depression Scale (SDS) [25] at baseline and at day 3, 7, 14, and 21. The secondary outcome measures included treatment response, defined as ≥50% reduction at endpoint from baseline on HAMD-17, and remission, defined as an endpoint HAMD-17 score of ≤7. Safety and tolerability were assessed using the Treatment Emergent Symptom Scale (TESS) [19], in which adverse events were recorded at each visit, including their date and time of onset, duration, severity, relationship to intervention, and the action taken.

Both patients and raters were blind to the treatment allocation. A training workshop with video materials was conducted for raters who might be involved in clinical assessments. An interrater reliability coefficient (κ value) of >0.80 was achieved after the completion of training workshop. In this study, all assessments were completed by the same rater (J.L.).

The credibility of n-EA and DCEAS procedure was evaluated based on Fink et al. method [26] by asking the patients: “As we informed you that you had an equal chance of receiving sham or active acupuncture treatment, which do you think you had received?”

### Statistical analysis

Based on our recent meta-analysis, a sample size of 70 patients (n = 35 per group) could provide approximately 80% power to detect an estimated difference in HAMD-17 score of 3 points, with α set at 0.05 and an estimated standard deviation of 4.5 at the endpoint of 3-week treatment [5].

Efficacy analyses were performed on the intention-to-treat population, defined as participants who completed baseline and at least one evaluation after treatment. Since measure time points were not balanced, a linear mixed-effects model was preferably applied to compare treatment outcomes (HAMD-17, CGI-S and SDS) over time between the two groups. The model was established using time and group for categorical fixed factors and random intercepts with scaled identity covariance matrix. Subject's age, gender, duration of the illness, number of relapse, baseline HAMD-17, tolerability and credibility for acupuncture procedure were treated as covariates. Between-group differences at each measure time point were examined using Student *t*-test. The data was expressed as mean with 95% confidence interval (95% CI). Student *t* test was used to compare continuous baseline variables between the two groups. Categorical variables, including categorical baseline variables, response and remission rates, incidence of adverse events, treatment compliance, and credibility, were analyzed using Chi-square (χ^2^) test or Fisher exact test if one or more expected frequencies were less than 5. Statistical significance was defined as a two-sided *P*<0.05. The analyses were performed with SPSS version 16 software (Chicago, IL, USA).

## Results

### Disposition and characteristics of patients

**Figure 2:**
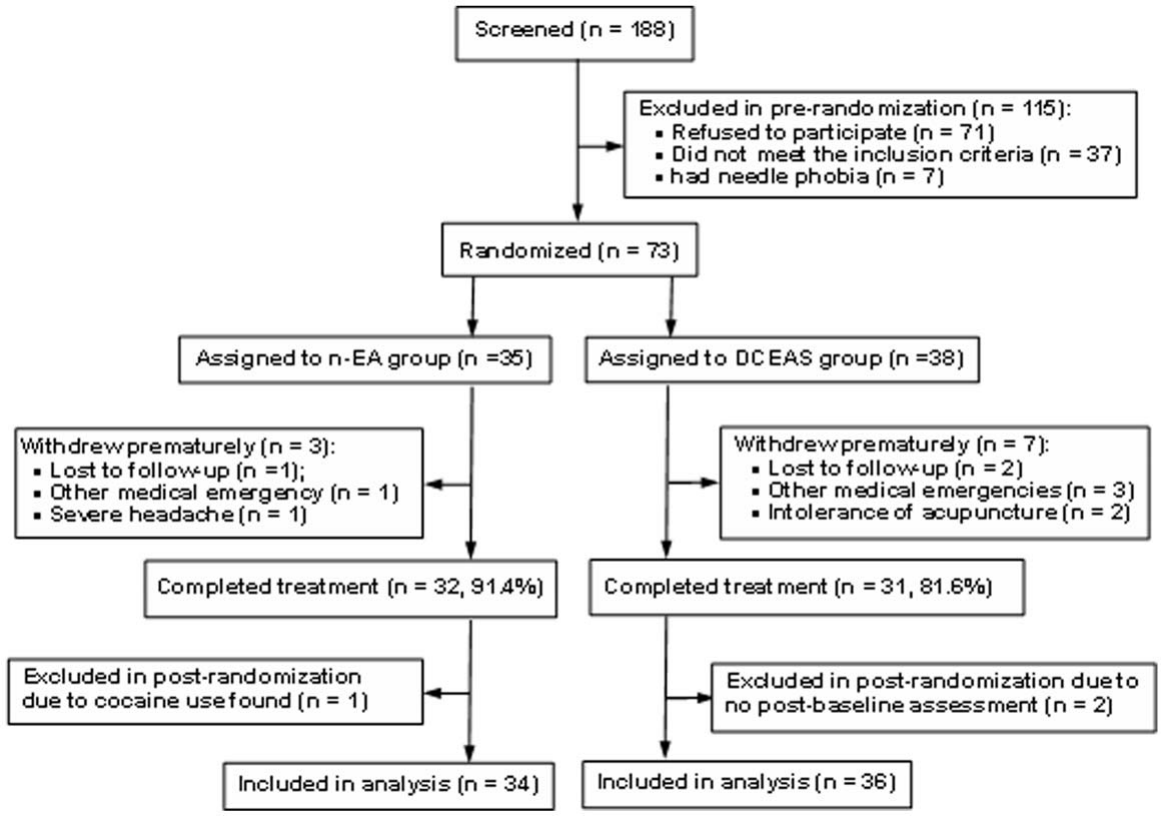
Flowchart of screening and patient recruitment. n-EA, noninvasive electroacupuncture; DCEAS, dense cranial electroacupuncture stimulation.

Of 188 outpatients referred by psychiatrists for screening, 73 eligible patients were randomly assigned to n-EA (n = 35) and DCEAS (n = 38) group; while 63 (86.3%) of them completed the 3-week assessment. One patient in n-EA group was excluded from analysis, because she was later found to have cocaine use in the past year. Two patients in DCEAS group were excluded from analysis due to a lack of post-baseline assessment. Seventy patients (34 in n-EA and 36 in DCEAS) were included in data analysis (Fig. 2).

Baseline characteristics of patients are summarized in Table 1. The proportion of females assigned to n-EA group was significantly higher than that in DCEAS group (97.1% vs. 69.4%, *P* = 0.006, Chi-square test). Other baseline variables were similar in the two groups. Nearly 93% (65/70) patients had experienced relapses and 66% (46/70) patients had acupuncture treatment previously. There were only 18.6% (13/70) of patients receiving psychotropic medication when entering the study. The compliance with acupuncture and FLX treatment was nearly 95% in the two groups.

**Table 1:** Baseline characteristics of patients.

Variables	n-EA(n = 34)	DCEAS(n = 36)	*P* values (*t* or χ^2^ test)
Female, n (%)	33 (97.1)	25 (69.4)	0.006
Age (yrs)^a^	48.2±9.8	46.3±9.9	0.414
Duration of MDD (yrs)^a^	7.3±7.1	7.9±8.0	0.744
No. of previous depressive episodes^a^	3.6±4.4	4.9±6.1	0.332
No. (%) of patients with first-onset MDD	3 (8.8)	2 (5.5)	0.669
No. (%) of patients with previous psychiatric admission	8 (23.5)	7 (19.4)	0.901
No. (%) of patients with family members having mental illnesses.	9 (26.5)	13 (36.1)	0.800
No. (%) of patients with previous acupuncture treatment^b^	22 (64.7)	24 (66.7)	0.937
No. (%) of patients receiving psychotropic medications at study entry^c^	6 (17.6)	7 (19.4)	0.909
SSRIs	3	3	
SNRIs	1	1	
Mood stabilizers	1^d^	1	
Benzodiazepines	2	2	
Baseline HAMD-17 score^a^	23.1±3.6	23.9±3.8	0.321
Baseline CGI-S^a^	4.3±0.5	4.4±0.5	0.760
Baseline SDS score^a^	40.6±14.5	41.9±14.0	0.704

a Continuous data are expressed as mean ± SD.

b Auricular acupuncture was included.

c The use of medications did not exceed one week.

d One patient received a combination of SNRIs and mood stabilizers.

n-EA, noninvasive electroacupuncture; DCEAS, dense cranial electroacupuncture stimulation; MDD, major depressive disorder; SSRIs, selective serotonin re-uptake inhibitors; SNRIs, Serotonin–norepinephrine reuptake inhibitors; HAMD, 17-item Hamilton Rating Scale for Depression; CGI-S, Clinical Global Impression-Severity; SDS, Self-rating Depression Scale.

### Efficacy

**Figure 3:**
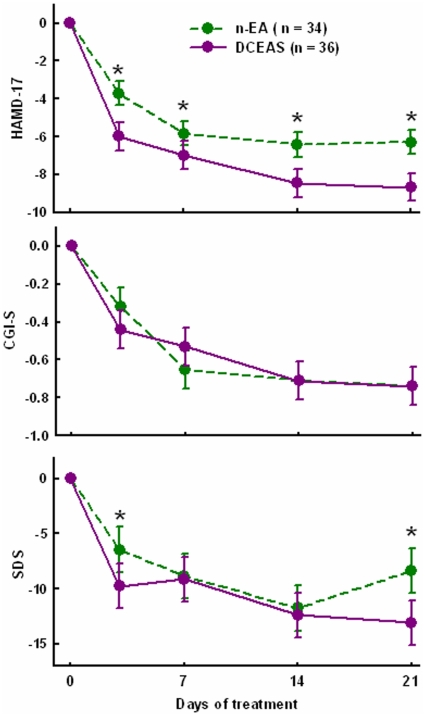
Mean changes from baseline in score on the 17-item Hamilton Rating Scale for Depression (HAMD-17), Clinical Global Impression-Severity (CGI-S) and Self-rating Depression Scale (SDS). Data are expressed as mean with 95% confidence interval (95% CI). * *P*≤0.037: between-group comparison using Student *t*-test.

Changes from baseline in score on HAMD-17, CGI-S, and SDS over time are illustrated in Table 2 and Fig. 3. The analyses based on linear mixed-effects model revealed highly linear correlations between measure time points and changes from baseline in score on HAMD (*r* ^2^ = 0.497 in n-EA group and *r* ^2^ = 0.531 in DCEAS group, *P*<0.0001), CGI (*r* ^2^ = 0.400 in n-EA group and *r* ^2^ = 0.381 in DCEAS group, *P*<0.0001), and SDS (*r* ^2^ = 0.192 in n-EA group and *r* ^2^ = 0.248 in DCEAS group, *P*<0.0001). There were significant differences in the slope and/or intercept between n-EA and DCEAS groups on HAMD (*F* = 5.938, df = 1,336, *P* = 0.015) and SDS (*F* = 5.885, df = 1,336, *P* = 0.016), but not CGI (*F* = 232, df = 1,336, *P* = 0.631). Between-group comparisons further revealed that DCEAS-treated patients had a significantly greater reduction in scores on HAMD-17 compared to patients receiving n-EA procedure at Day 3 through Day 21 (*P*≤0.025). The significantly greater reduction was also observed in SDS scores at day 3 (*P* = 0.037) and day 21 (*P* = 0.004).

**Table 2:** Changes in score on depression scales from baseline in MDD patients.

Variables	n-EA (n = 34)(95% CI)	DCEAS (n = 36)(95% CI)	Between-group difference (95% CI)	Overall *P* value^a^	Between-group *P* value^a^
**HAMD-17**				0.015	
Day 3	−3.71 (−4.34–−3.06)	−5.97 (−6.71–−5.23)	2.27 (1.29–3.25)		0.000
Day 7	−5.82 (−6.46–−5.18)	−6.97 (−7.71–−6.23)	1.15 (0.17–2.13)		0.025
Day 14	−6.41 (−7.05–−5.77)	−8.44 (−9.18–−7.70)	2.03 (1.05–3.01)		0.000
Day 21	−6.27 (−6.90–−5.62)	−8.66 (−9.39–−7.91)	2.39 (1.41–3.37)		0.000
**CGI-S**				0.631	
Day 3	−0.32 (−0.42–−0.22)	−0.44 (−0.54–−0.34)	0.12 (−0.03–0.27)		0.116
Day 7	−0.65 (−0.75–−0.55)	−0.53 (−0.63–−0.43)	0.12 (−0.03–0.27)		0.116
Day 14	−0.71 (−0.81–−0.61)	−0.71 (−0.81–−0.61)	0.00 (−0.15–0.15)		1.000
Day 21	−0.74 (−0.84–−0.64)	−0.74 (−0.84–−0.64)	0.00 (−0.15–0.15)		1.000
**SDS**				0.016	
Day 3	−6.44 (−8.48–−4.40)	−9.76 (−12.03–−7.49)	3.32 (0.26–6.38)		0.037
Day 7	−8.82 (−10.86–−6.78)	−9.12 (−11.39–−6.85)	0.30 (−2.76–3.36)		0.851
Day 14	−11.74 (−13.78–−9.70)	−12.38 (−14.65–−10.11)	0.64 (−2.42–3.70)		0.679
Day 21	−8.38 (−10.42–−6.34)	−13.06 (−15.33–−10.79)	4.68 (1.62–7.74)		0.004

a Overall and between-group *P* values were obtained from linear mixed-effects model analysis and student *t*-test, respectively.

MDD, major depressive disorder; n-EA, noninvasive electroacupuncture; DCEAS, dense cranial electroacupuncture stimulation; 95% CI, 95% confidence interval; HAMD-17, 17-item Hamilton Rating Scale for Depression; CGI-S, Clinical Global Impression-Severity; SDS, Self-rating Depression Scale.

The response rate in DCEAS group was not significantly different from that in n-EA group (19.4% (7/36) vs. 8.8% (3/34), *P* = 0.308, Fisher Exact test). The remission rate was also similar in the two groups (2.7% (1/36) vs. 2.9% (1/34), *P* = 0.998, Fisher Exact test).

The average dose of FLX in DCEAS group was similar to that in n-EA group (23.01±3.2 mg/day (mean ± SD) vs. 23.4±2.4 mg/day, *P* = 0.599, *t*-test).

### Safety and tolerability

Adverse events occurred in at least 5% of the patients in either group are listed in Table 3. No significant differences in the incidence of any adverse events were found between the two groups. There were 20.6% (7/34) of patients who felt uncomfortable in n-EA procedure, but not significantly different from 38.9% (14/36) in DCEAS-treated patients (χ^2^ = 1.985, df = 1, *P* = 0.159). Two patients in DCEAS group discontinued due to intolerance of acupuncture stimulation.

**Table 3:** Adverse events occurred in at least 5% of patients in either group.

Event	n-EA (n = 34)	DCEAS (n = 36)	χ^2^	*P* value
Dizziness	15 (44.1)	11 (30.6)	0.858	0.354
Tiredness	10 (29.4)	15 (41.7)	0.672	0.412
Nausea	10 (29.4)	10 (27.8)	0.013	0.910
Excessive sweating	9 (26.5)	6 (16.7)	1.403	0.236
Headache	8 (23.5)	10 (27.8)	0.018	0.894
Transient tachycardia	8 (23.5)	9 (25.0)	0.018	0.892
Insomnia	7 (20.6)	9 (25.0)	0.024	0.877
Uncomfortable for needling sensation	7 (20.6)	14 (38.9)	1.985	0.159
Vomiting	4 (11.8)	3 (8.3)		0.706^a^
Unsteadiness	2 (5.9)	6 (16.7)		0.266^a^
Somnolence	2 (5.9)	6 (16.7)		0.266^a^

a *P* values were calculated from Fisher Exact test.

n-EA, noninvasive electroacupuncture; DCEAS, dense cranial electroacupuncture stimulation.

### Credibility of sham and DCEAS procedure

There was no significant difference in the credibility rating between the two groups, with 45.5% (15/33) of patients treated with n-EA perceiving to have received DCEAS, while 23.5% (8/34) of patients in DCEAS believed to have n-EA treatment (χ^2^ = 2.665, *P* = 0.103).

## Discussion

The present study demonstrated that DCEAS intervention is effective in augmenting the antidepressant efficacy of FLX in the treatment of moderate and severe MDD. While the patients in the two groups had received similar FLX doses during the study, DCEAS-treated patients exhibited greater improvement on depressive symptoms, as indicated with the significant greater reduction of HAMD-17 and SDS score at most measure time points, although the magnitude of the reduction of SGI-S score, the response and remission rates were not different in the two groups. Moreover, the greater reduction of both HAMD-17 and SDS was observed as early as at day 3 after the first session of acupuncture treatment. Similar result was also present at endpoint of three weeks of DCEAS intervention. These data suggest that DCEAS intervention produces a rapid effect in alleviating depressive symptoms in both clinician-rated (HAMD-17) and self-rated (SDS) measures of depression. In addition, there were only two DCEAS-treated patients who discontinued due to intolerance of acupuncture. DCEAS intervention did not increase the incidence of any adverse events compared to n-EA control procedure, suggesting that DCEAS is a tolerable and safe stimulation mode.

While the current study showed the superior antidepressant efficacy of DCEAS over n-EA procedure when combined with FLX, several similar trials failed to demonstrate the superior effects of active acupuncture regimens in reducing depressive symptoms compared to sham and placebo acupuncture regimens [27]–[29]. This has raised the argument that the antidepressant benefits of acupuncture observed may be derived from placebo effects rather than physiological mechanisms [28]. Nevertheless, the present study revealed no significant difference in the credibility of the control and DCEAS procedures, with nearly 46% of n-EA-treated patients who perceived to have received active procedure, while 24% of DCEAS-treated patients perceived the control procedure, suggesting that the non-inserted needling stimulation used in the present study for a control procedure was valid and acceptable. In fact, previous studies have well demonstrated the high credibility of the non-inserted needle device [26]. Therefore, it was unlikely that the antidepressant benefits of DCEAS observed in the present study were derived from placebo effects.

There are two possible explanations for negative results in the previous acupuncture studies of major depression [27]–[29]. Firstly, unlike the present study that used the non-inserted control procedure, the previous studies needled at non-meridian-based acupoints which are located at a certain distances (usually 1–3 cm) from the meridian-based acupoints [27]–[29]. Although there seems to be some differences in the histological profile between the meridian- and non-meridian-based acupoints [30], it might be difficult to differentiate the physiological responses induced by stimulation at the two types of acupoints. Second, relatively few acupoints were used in the previous studies [27]–[29]. This may result in inadequacy of acupuncture stimulation which is believed to be an important factor associated with negative results of acupuncture trials [31].

DCEAS was developed mainly based on a neurobiological rationale. It is well documented that the forehead acupoints innervated by the trigeminal sensory pathway have intimate collateral connections with the brainstem reticular formation, in particular the dorsal raphe nucleus (DRN) [32], [33] and the locus coeruleus (LC) [34]–[37]. The latter two brain structures are the major resources of 5-HT and NA neuronal bodies, respectively, sending diffuse projections to subcortical and cortical areas, including the prefrontal cortex and the amygdala known to be heavily involved in the pathogenesis of depressive disorders [38]. A large body of evidence confirms that the brainstem 5-HT and NA neuronal systems play a pivotal role in acupuncture modulation of multiple brain functions, including pain, emotion, sleep, and visceral information processing [8]–[10], [39]. Neuroanatomical and neurophysiological studies have demonstrated that electroacupuncture stimulation increases the expression of 5-HT in the DRN [40] and suppresses the stress-induced increase in neuronal activities of the LC [41], [42]. Neuroimaging studies have also shown that electroacupuncture stimulation is capable of directly modulating the activity of the emotion processing-related brain regions [8]. Through the intimate collateral connection from the trigeminal sensory pathway to the brainstem 5-HT and NA neuronal systems, the needling of the forehead acupoints with subsequent electrical stimulation could robustly elicit afferent acupuncture signals via biophysical and biochemical reactions at local acupoints and, in turn, efficiently modulates central 5-HT and NA neuronal functions [9], [10]. On the other hand, like other noninvasive brain stimulation therapies, such as transcutaneous electrical nerve stimulation (TENS), repetitive transcranial magnetic stimulation (rTMS), and electroconvulsive therapy (ECT) [43], [44], electrical stimulation was also directly delivered on the scalp in both control and DCEAS procedure. This may elicit a transcutaneous and/or transcranial effect; however, such effect would likely be minimal as the stimulation intensity used in both n-EA control and DCEAS procedures was generally much lower than rTMS, ECT, and most TENS [43], [44]. Therefore, we have reason to believe that the antidepressant efficacy of DCEAS observed in the present study should be derived mainly from the biophysical and biochemical effects produced in needling with subsequent electrical stimulation [45]. This also could explain the superior therapeutic efficacy of DCEAS over n-EA control procedure.

Several limitations of the present study should be noticed. First, the study was conducted in a female-dominated sample with a significant difference in the proportion of female subjects between the two groups. Epidemiological evidence suggests that women are more likely to use complementary and alternative medicine (CAM) and have a higher degree of confidence in CAM efficacy and safety [46]. Whether similar therapeutic effects of DCEAS could be achieved in male patients needs to be further investigated. In addition, as the study was conducted in a single-blind manner, effects mediated by non-blinded acupuncturists could not be excluded. Recently, a well-demonstrated control method for single-blind condition has been introduced and could be considered in future studies [47]. Second, although DCEAS achieved a clinically meaningful, over 2-fold difference in the response rate than the control procedure (19.4% vs. 8.8%), the difference did not reach statistical significance level. This may be due to the relatively low response rates in the two groups when compared to other similar trials, with response rates of 22–80% for active acupuncture and 39–78% for sham or placebo-treated groups [27]–[29]. The relatively low response rate observed in the present study appears to be related to the short-term (3-week) treatment. As long-term effects of DCEAS were not evaluated in this study and a majority of depressed patients may be required for long-term treatment [48], long-term antidepressant efficacy of DCEAS may deserve to be further investigated. Finally, although we measured patients' platelet 5-HT contents at baseline and posttreatment (data not shown in this report), no significant changes in platelet 5-HT parameters were observed in DCEAS-treated patients compared to patients treated with n-EA, suggesting that acupuncture may have least effects on non-neuronal 5-HT systems. Evaluation of DCEAS effects in the brain 5-HT neuronal system may help gain new insight into central mechanisms responsible for DCEAs effects.

Collectively, the present study demonstrates that DCEAS is a safe and effective intervention in augmenting the antidepressant efficacy in the early phase of SSRI treatment. As patients with moderate and severe major depression have a higher risk of suicide and the worsening of symptoms in the early phase of SSRI treatment, DCEAS can be considered as an additional treatment option. The present study guarantees a larger-scale, multi-site trial to further determine the effectiveness of DCEAS as a viable and safe non-pharmacological augmentation for depressive disorders.

## Supporting Information

Checklist S1 CONSORT Checklist.
(PDF)

Protocol S1 Trial Protocol.
(PDF)
