# Correction: Dense Cranial Electroacupuncture Stimulation for Major Depressive Disorder—A Single-Blind, Randomized, Controlled Study

**DOI:** 10.1371/annotation/b27d20b4-f41c-47af-b19f-a0278c993a2d

**Published:** 2012-02-07

**Authors:** Zhang-Jin Zhang, Roger Ng, Sui Cheung Man, Tsui Yin Jade Li, Wendy Wong, Qing-Rong Tan, Hei Kiu Wong, Ka-Fai Chung, Man-Tak Wong, Wai-Kiu Alfert Tsang, Ka-chee Yip, Eric Ziea, Vivian Taam Wong

There was an error in Roger Ng's affiliation. The correct affiliation is 2 Department of Psychiatry, Kowloon Hospital, Hong Kong, China. 

