# Supplementary material for: Dense Cranial Electroacupuncture Stimulation for Major Depressive Disorder—A Single-Blind, Randomized, Controlled Study
Source: PLoS One. 2012 Jan 6;7(1):e29651. doi: 10.1371/journal.pone.0029651 (PMC3253099; doi:10.1371/journal.pone.0029651)
Supplement: Protocol S1 — Trial Protocol. (PDF) [file pone.0029651.s002.pdf]

Study protocol

A. Title:

A single-blind, randomised, sham controlled study of electroacupuncture in accelerating the onset of antidepressant action of selective serotonin reuptake inhibitors via serotonergic mechanisms

B. Introduction

Depression is a worldwide mental health problem, with a lifetime prevalence of about 20%. In Hong Kong, there are about 400-600 thousands of depressed patients with major depressive disorder (MDD) annually. However, the currently available antidepressant treatments, represented by selective serotonin reuptake inhibitors (SSRIs), are incomplete and unsatisfactory. The most apparent is the delay in the onset of action of SSRIs, which has hampered the use of this class of drugs. This is not only because the suffering of patients prolonged after commencement of treatment, but also because it leaves the patients exposed to great risk of suicide. Moreover, adverse effects which are frequently at their worst during the initial phases of treatment often result in discontinuation. Thus, the exploration of faster acting antidepressant strategies is greatly needed.

Serotonergic (5-HT) system plays a principal role in the mechanisms responsible for the slow onset of action of SSRIs. In initial phases of SSRI treatment, increased 5-HT levels in the synaptic gaps produced by reuptake blockade activate negative feedback via autoreceptors in the raphe 5-HT neurons (5-HT_{1A}) and 5-HT projection forebrain areas (5-HT_{1B}), resulting in a decrease in the net amount of the extracellular 5-HT by suppressing neuronal cell firing and terminal release. Repeated administration of SSRIs, however, results in the desensitization of autoreceptors, which enables 5-HT neurons to recover cell firing and leads to an increase in the extracellular 5-HT to a level higher than seen in initial treatment. Therefore, it is believed that the blockade of the negative feedback potentiate the 5-HT increase produced by SSRIs and might serve to accelerate the clinical effects of SSRIs.

Numerous studies have shown beneficial effects of electroacupuncture (EA) as additional therapy in depressed patients and the most apparent is that EA intervention remarkably shortens the latency of clinical response to SSRIs. This effect has also been confirmed in our meta-analysis and preliminary study, suggesting that EA intervention can accelerate the onset of antidepressant action. On the other hand, a large body of evidence confirms that neural effects of EA (e.g., analgesic effects) are strongly associated with the activation of the raphe 5-HT neurons. Indeed, electrophysiological and neurochemical studies have revealed that EA stimulation directly facilitates the raphe 5-HT neuronal body firing and 5-HT release in the raphe nuclei itself and the forebrain areas of mood processing. Moreover, EA stimulation inhibits the activity of 5-HT_{1A} receptors and modulates 5-HT_{1B} receptor and 5-HT transporter function. Our preliminary studies in patients with MDD also demonstrated that the addition of EA to paroxetine (PRX), an SSRI, significantly elevates plasma 5-HT levels in the initial phase of treatment (at week 2) and reduces the expression of platelet 5-HT_{1A} receptors, but does not change platelet 5-HT levels, 5-HT uptake and turnover ratios, while PRX treatment alone results in an increase in the 5-HT_{1A} expression.

These observations have led us to hypothesize that EA intervention could accelerate the clinical response to SSRIs in patients with MDD and this acceleration may be achieved

through its potentiation of 5-HT release by inhibiting autoreceptor (5-$HT_{1A/1B}$) activities, uptake, and turnover. To test this hypothesis, we propose a single-blind, randomised, sham controlled study to determine relationships between the accelerating effects of EA and platelet 5-HT activities in the course of EA intervention in combination with SSRIs in patients with MDD.

The proposed project will open a new avenue towards developing faster acting antidepressant strategies from alternative medicines and provide scientific evidence to support EA as an "accelerator" used for the treatment of MDD and other psychiatric disorders associated with 5-HT. the proposed project will also bridge gaps between traditional and contemporary medicines. Two apparently innovative aspects of this application may deserve to be addressed: the development of faster acting antidepressant strategies from alternative medicines is an innovative idea and the hypothesis that 5-HT system plays a central role in antidepressant-accelerating effects of acupuncture is novel and original.

C. Aims and hypothesis

The central theme of this proposed study is to test the hypothesis that EA intervention could accelerate the clinical response to SSRIs in patients with MDD. Meanwhile, we also would like to determine whether the accelerative effects of EA are achieved through its potentiation of 5-HT release by inhibiting autoreceptor (5-$HT_{1A/1B}$) activities, uptake, and turnover. To test these hypotheses, a single-blind, randomised, sham controlled study is proposed to detect differences in the clinical and platelet 5-HT responses between MDD patients treated with fluoxetine (FLX, an SSRI) in combination with active and sham EA intervention. The three aims are as follows:

(1) To determine whether the patients in active EA intervention could yield significantly greater improvements on depressive symptoms, clinical response, remission rates, and the latency to clinical response than in sham EA intervention following 3 weeks of treatment;
(2) To compare the effects of sham and active EA intervention in combination with FLX on platelet 5-HT levels, uptake, turnover, 5-$HT_{1A/1B}$ receptors and 5-HT transporter (SERT) activities; and
(3) To determine correlations between clinical measures and platelet 5-HT indices.

D. Plan of investigation

<u>*Outline:*</u> This is a 3-week, single-blind, randomised, sham controlled study. A total of 70 patients with a primary diagnose of MDD will be recruited. Under single-blind condition, patients will be randomly assigned to FLX (10-30 mg/day) combined with 12 sessions of active or sham EA for 3 weeks. EA intervention of each session is conducted by electrically stimulating six pairs of acupoints for 30 min. The primary efficacy is measured using the 17-Item Hamilton Depression Rating Scale (HAMD-17), Clinical Global Impression-Severity Scale (CGI-S), and Self-Rating Depression Scale (SDS). The measures for the secondary efficacy include clinical response, remission rates, and the latency to the clinical response. Two blood samples will be collected from patients at baseline and at week 2 for the measurements of platelet 5-HT, 5-HIAA, SERT, and 5-$HT_{1A/1B}$ receptors using HPLC, binding assay, and immunoblotting methods. One blood sample will be collected from an additional group of healthy subjects (n = 12) as controls.

Subjects: Both in- and out-patients who meet all the following criteria will be eligible for the study: (1) either gender aged 25-65 years; (2) have MDD diagnosed as the DSM-IV; and (3) scores on the 17-item HAMD and CGI-S are at least 18 and 4 points, respectively. Patients who meet one of the following criteria are excluded from the study: (1) unstable medical conditions; (2) have suicidal attempts or aggressive behavior; (3) previously experienced manic, hypomanic, or mixed episode; or (4) immediate family members were or are diagnosed for bipolar or psychotic disorders; (5) treatment with investigational drugs in past 6 months; (6) alcoholism or drug abuse in past 1 year; (7) have needle phobia; or 8) had experienced acupuncture or electroacupuncture.

Since the pathogenesis of bipolar depression and its response to SSRIs are different from unipolars, it may need to be screened out. Bipolar disorder has been found to be strongly associated with first manic, hypomanic, or mixed episode, bipolar family history, and early age at onset of first major depressive episode (before 21 years). We therefore set entry age at least 25 years and the Exclusive Criteria 2-4. In addition, since previous experience of acupuncture may interfere with the blindness of EA, the subjects who have experienced acupuncture previously will be excluded.

Estimation of sample size: In order to ensure the power sufficient to detect EA effects, we perform estimations of sample size based on response rate and HAMD score, respectively.(I) Based on our preliminary data, showing nearly 36%-43% between the two groups at week 2-3, the proposed study is intended to detect a minimum 40% increase in clinical response rate following 2 weeks of EA intervention, with a power (1-β) of 80% and a two-tailed level of α = 0.05. Based on these assumptions, 28 patients per group are needed. (2) Based on our initial data, EA treatment could yield a 6-point reduction in HAMD score at week 2 compared to SSRI alone. The study is then proposed to detect a minimum 6-point difference between the two treatment groups in change in HAMD score at week 2 from baseline with a power (1-β) of 80% and a two-tailed level of α = 0.05. Based on these assumptions, 28 subjects per group are needed. The two estimations suggest the same sample size. Therefore, we will recruit 70 patients for the study when 20% dropouts are expected.

Randomization and blind assignment: Patients will be randomized to assign sham or active EA intervention in blocks of 2 subjects with strata of gender and age. Randomination will be processed by the PI using random digital table.

The study is in single-blinded manner, i.e., patients are not aware of either active or sham acupuncture treatment (see below). Apart from sham acupuncture procedure (see below), additional measures will be taken to ensure blinding condition: (1) Since all acupuncture points used in the proposed study (see below) are located in the head, patients in general could not visually detect the acupuncture procedures. Despite this, we will ask patients to close their eyes during acupuncture treatments; (2) Clinical assessors will be blind to acupuncture treatment condition of patients; (3) To avoid patients' communication and observations each other, we will use curtains to separate each bed during acupuncture treatment.

Treatment procedures: Both groups of patients will receive orally administered FLX for 3 weeks. FLX dose will be given at 10 mg/day for the first 3 days, 20 mg/day from the 4th day to 7th, and 30 mg/day from the second week and thereafter. This dosing regimen of FLX has been reported to yield optimal clinical outcomes. The choice of FLX is because this SSRI is the most commonly used antidepressant in Hong Kong psychiatry practices and, similar to PRX, the slow onset of FLX action has been well demonstrated
